# Principal component analysis for the comparison of metabolic profiles from human rectal cancer biopsies and colorectal xenografts using high-resolution magic angle spinning ^1^H magnetic resonance spectroscopy

**DOI:** 10.1186/1476-4598-7-33

**Published:** 2008-04-25

**Authors:** Therese Seierstad, Kathrine Røe, Beathe Sitter, Jostein Halgunset, Kjersti Flatmark, Anne H Ree, Dag Rune Olsen, Ingrid S Gribbestad, Tone F Bathen

**Affiliations:** 1 Department of Medical Physics, Rikshospitalet-Radiumhospitalet Medical Center, 0310 Oslo, Norway; 2 Faculty of Health, Buskerud University College, 3007 Drammen, Norway; 3 Department of Radiation Biology, Rikshospitalet-Radiumhospitalet Medical Center, 0310 Oslo, Norway; 4 Department of Circulation and Medical Imaging, Norwegian University of Science and Technology (NTNU), 7489 Trondheim, Norway; 5 Department of Laboratory Medicine, Children's and Women's Health, Norwegian University of Science and Technology (NTNU), 7489 Trondheim, Norway; 6 Department of Surgical Oncology, Rikshospitalet-Radiumhospitalet Medical Center, 0310 Oslo, Norway; 7 Department of Tumor Biology, Rikshospitalet-Radiumhospitalet Medical Center, 0310 Oslo, Norway; 8 University of Oslo, 0316 Oslo, Norway; 9 Institute for Cancer Research, Rikshospitalet-Radiumhospitalet Medical Center, 0310 Oslo, Norway

## Abstract

**Background:**

This study was conducted in order to elucidate metabolic differences between human rectal cancer biopsies and colorectal HT29, HCT116 and SW620 xenografts by using high-resolution magnetic angle spinning (MAS) magnetic resonance spectroscopy (MRS) and for determination of the most appropriate human rectal xenograft model for preclinical MR spectroscopy studies. A further aim was to investigate metabolic changes following irradiation of HT29 xenografts.

**Methods:**

HR MAS MRS of tissue samples from xenografts and rectal biopsies were obtained with a Bruker Avance DRX600 spectrometer and analyzed using principal component analysis (PCA) and partial least square (PLS) regression analysis.

**Results and conclusion:**

HR MAS MRS enabled assignment of 27 metabolites. Score plots from PCA of spin-echo and single-pulse spectra revealed separate clusters of the different xenografts and rectal biopsies, reflecting underlying differences in metabolite composition. The loading profile indicated that clustering was mainly based on differences in relative amounts of lipids, lactate and choline-containing compounds, with HT29 exhibiting the metabolic profile most similar to human rectal cancers tissue. Due to high necrotic fractions in the HT29 xenografts, radiation-induced changes were not detected when comparing spectra from untreated and irradiated HT29 xenografts. However, PLS calibration relating spectral data to the necrotic fraction revealed a significant correlation, indicating that necrotic fraction can be assessed from the MR spectra.

## Background

Animal models are frequently used in the study of complex human diseases. With the use of new technology and the availability of transgenic animals, molecular mechanisms of several diseases can be explored. In order for an animal model to serve as a useful model for human disease, the modeled disease must be similar in etiology and function to the human equivalent. Man-mouse xenografts have been widely used to assess the therapeutic effect of carcinostatic drugs on human malignant cells [1-3]. However, many drugs that show anti-tumor activity in subcutaneous (s.c.) xenograft models have shown disappointing results in the clinics [4,5]. Characterization of tissue at the molecular level will elucidate differences and similarities between metabolic profiles of human rectal cancer and xenografts. Knowledge of the metabolic profile of a xenograft can be important for its predictive value as tumor model in preclinical biomedical research.

*In vivo *proton magnetic resonance spectroscopy (^1^H MRS) is used to elucidate biochemical profiles of tissues in a number of disorders [6-8]. Limitations on the strength and inherent inhomogeneity of the magnetic fields restrict these studies to a handful of high-concentration metabolites. *Ex vivo *^1^H nuclear magnetic resonance (NMR) spectroscopy of excised tissue generate spectra that enable separation and relative quantification of the majority of metabolites in the tissue. But, due to the molecular constraints of semi-solids, ordinary high resolution (HR) MRS of excised tissue results in broadening of the metabolite peaks in the MR spectra. As dipole couplings and chemical shift anisotropy both are scaled according to the term (3cos^2^*θ*-1), positioning the sample at the so-called magic angle of 54.7° to the magnetic field and then spinning it rapidly about its own axis, reduce these interactions [9].

The first applications of HR MAS MRS on human tissue specimens date back to 1997 when Moka *et al*. [10] applied it to human kidney tissue, Millis *et al*. [11] to human lipoma and liposarcoma tissue and Cheng *et al*. [12] to human brain samples. HR MAS MR spectroscopy has later been used in numerous studies of both healthy and affected tissue, with the application to brain, breast and prostate cancer tissue [13-15]. HR MAS MRS has a potential to become a supplement in clinical diagnostics [16,17]. To the authors knowledge, there are no studies reported where HR MAS MRS has been used for biochemical characterization of human colorectal tumor tissue.

The histological differences of tumors arising in mice compared to those in humans have been a major limitation of many mouse models. To select the appropriate xenograft model, it is also important to know the geno- and phenotypic differences between experimental animal xenografts and human tumor tissue. In order to determine the most appropriate human rectal cancer xenograft model for preclinical MR spectroscopy, we used principal component analysis for comparing the metabolic profile of human rectal tumor biopsies with three common colorectal cell lines using HR MAS MR spectroscopy. Furthermore, we investigated the changes in biochemical composition of HT29 xenografts following 15 Gy irradiation.

## Results

### Spectral assignment

**Figure 1:**
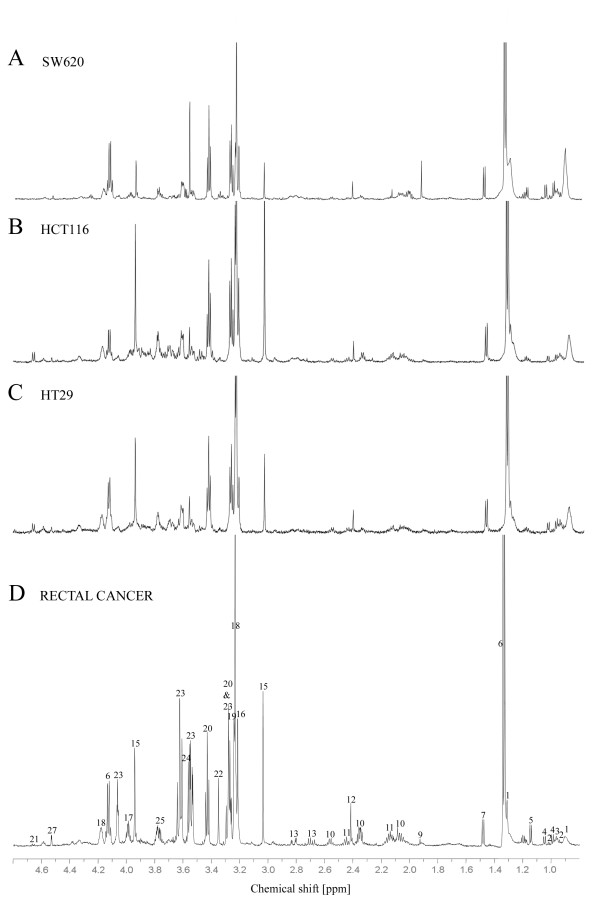
**Representative high-resolution proton spectra (4.7 - 0.5 ppm) of SW620 (A), HCT116 (B), HT29 (C), and a human rectal adenocarcinoma biopsy (D).** For display the spectral amplitude of the lactate doublet at 1.33 ppm have been cut. Assignments are given in Table 1.

**Figure 2:**
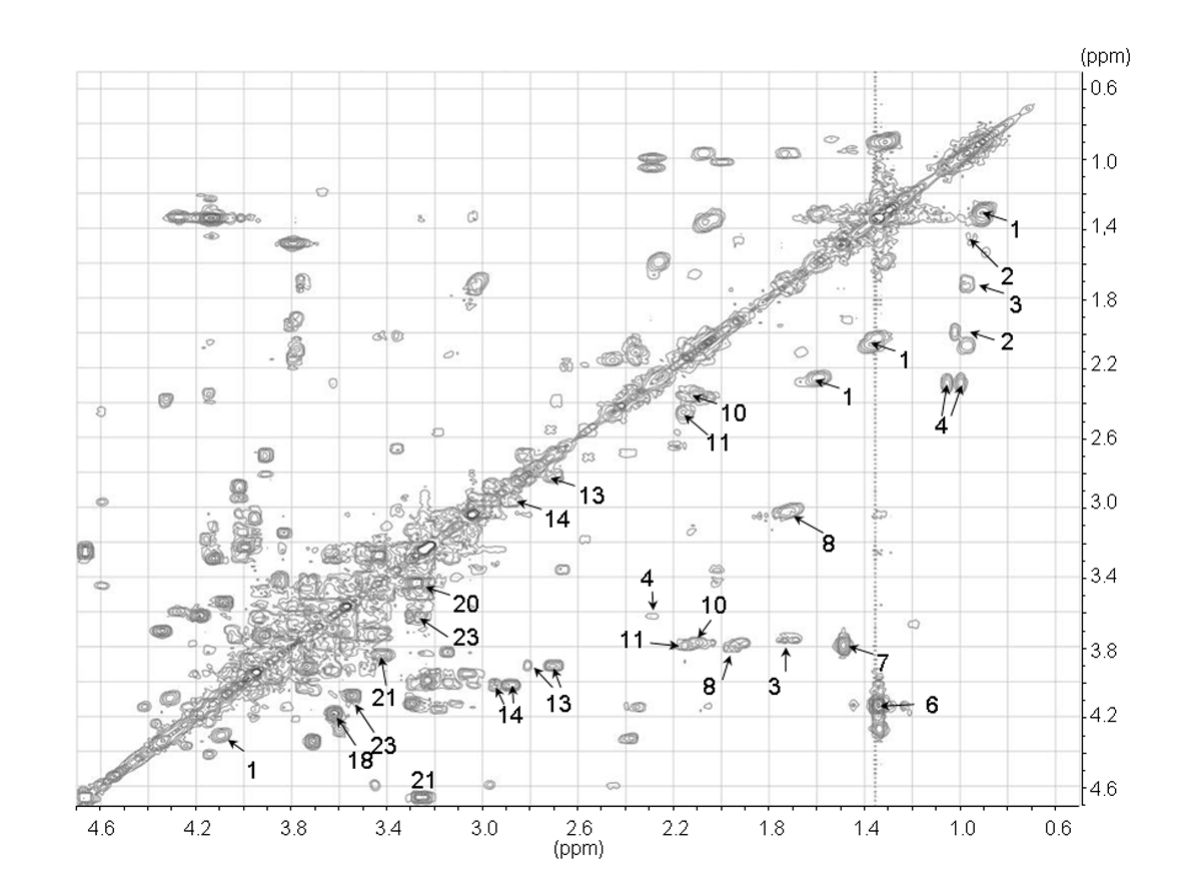
**Partial homonuclear correlated spectrum (COSY) of tissue from HT29 xenograft (spectral region 4.7 - 0.5 ppm).** The identified metabolites are denoted with labels according to Table 1.

Representative CPMG spin-echo HR MAS MR spectra of the colorectal xenografts SW620, HCT116 and HT29 are presented in Fig. 1 together with the spin-echo spectra from a human rectal adenocarcinoma biopsy. High spectral resolution of the one dimensional spin-echo spectra combined with reported data from the literature (18,19) and two dimensional proton-proton spectroscopy (COSY, Fig. 2) allowed identification and assignment of 27 different metabolites (Table 1) between 4.7 and 0.5 ppm. Amino acids, choline-containing compounds, creatine, inositols and lactate dominated the spin-echo spectra. Although lipids were the most dominant peaks in the single-pulse spectra, the metabolites were clearly visible also in the single-pulse spectra. In general, the HR MAS MR spectra of the colorectal xenografts and the human rectal cancer tissue contained the same metabolites and classification was based on differences between the relative amounts of the same metabolites. Glucose was either not detectable or appearing in low levels in HCT116, SW620 and human tissue samples. In HT29 xenografts however, glucose was detected in all samples with high levels in some of them. Beta-hydroxybutyrate was only detected in the human rectal biopsies.

**Table 1:** Chemical shift assignment.

Assigned number	Metabolite		Multiplicity	ppm
1	Fatty acids	C**H**_3_	t	0.89
		(C**H**_2_)_n_	m	1.31
		C**H**_2_CH_3_	m	1.35
		C**H**_2_CH_2_CO	m	1.59
		CH=CHC**H**_2_CH_2_	m	2.03
		-CH_2_-C**H**_2_-CO-	m	2.26
2	Isoleucine	δCH3	t	0.95
		γCH_3_	d	1.02
		γCH	m	1.46
		βCH	m	1.99
3	Leucine	δ'CH3	d	0.95
		δCH3	d	0.97
		βCH	m	1.71
		αCH	t	3.75
4	Valine	γCH_3_	d	0.99
		γ'CH_3_	d	1.04
		βCH	m	2.29
		αCH	d	3.62
5	Betahydroxybutyrate	CH_3_	d	1.18
6	Lactate	CH_3_	d	1.33
		CH	q	4.13
7	Alanine	βCH_3_	d	1.48
		αCH	q	3.79
8	Lysine	γCH_2_	m	1.48
		δCH_2_	m	1.73
		βCH_2_	m	1.92
		εCH_2_	t	3.02
		αCH_2_	t	3.79
9	Acetate	CH_3_	s	1.94
10	Glutamate	βCH2	c	2.11
		γCH_2_	t	2.34
		αCH	t	3.77
11	Glutamine	βCH2	c	2.14
		γCH2	c	2.45
		αCH	t	3.79
12	Succinate	(α,βCH_2_)	s	2.41
13	Aspartate	βCH	dd	2.71
		β'CH	dd	2.83
		αCH	dd	3.91
14	Tyrosine	βCH	dd	2.87
		β'CH	dd	2.96
		αCH	dd	4.02
15	Creatine	CH_3_	s	3.04
		CH_2_	s	3.94
16	Choline	N(CH_3_)_3_	s	3.22
17	Phosphoethanolamine	NCH_2_	dd	3.23
		OCH_2_	dd	3.99
18	Phosphocholine	N(CH_3_)_3_	s	3.23
		NCH_2_	dd	3.62
		OCH_2_	dd	4.18
19	Glycerophosphocholine	N(CH_3_)_3_	s	3.24
20	Taurine	N-CH_2_	t	3.24
		S-CH_2_	t	3.42
21	β-Glucose	C2H	t	3.26
		C5H	dd	3.42
		C6H	d	3.85
		C1H	d	4.64
22	*scyllo*-Inositol	(All Hs)	s	3.35
23	*myo*-Inositol	C5H	t	3.27
		C1H, C3H	dd	3.52
		C4H, C6H	t	3.62
		C2H	t	4.07
24	Glycine	αCH2	s	3.56
25	Amino acid residues	αCH	q	3.77
26	Triglycerides, glyceryl	1/2 C**H**_2_OCOR	m	4.09
		1/2 C**H**_2_OCOR	m	4.31
27	Ascorbic acid	C4H	d	4.53

Tentative ^1^H chemical shift assignment of HR MAS MR spectra obtained from human rectal cancer tissue and xenografts. Chemical shift referencing is relative to TSP. Peak multiplicity is given as s: singlet, d:doublet, dd: double doublet, t: triplet, q: quartet, m: multiplet or c: complex. Assigned numbers correspond to the numbering in Fig. 1 and Fig 2.

### Histology

Histological examination of HR MAS MRS analyzed tissue from non-irradiated samples revealed large differences in viable tumor cells, fibrosis and necrotic tissue (Table 2). Whereas necrotic areas were found in all cross-sections from xenografts, no necrosis was observed in human rectal cancer tissue samples. The necrotic fractions varied from 5 to 80% for the xenograft samples. However, one-way ANOVA showed no significant differences between the amounts of necrosis in the three untreated xenografts (p = 0.49).

**Table 2:** Tumor histology.

	**n**	**Tumor cells [%]**	**Necrosis [%]**	**Fibrosis [%]**
**SW620**	5	60 (30–70)	40 (10–60)	10 (0–20)
**HCT116**	7	70 (20–90)	10 (5–60)	20 (0–20)
**HT29**	8	65 (10–80)	15 (5–80)	20 (10–20)
**Rectal cancer biopsies**	10	35 (10–98)	0 (0-0)	28 (0–60)

Percentage viable tumor cells, necrosis and fibrosis in the HR MAS MRS analyzed tissue samples from untreated SW620, HCT116, HT29 and human rectal cancer biopsies. Results are given as median and range.

### Comparison of metabolic profiles of xenografts and human rectal cancer

**Figure 3:**
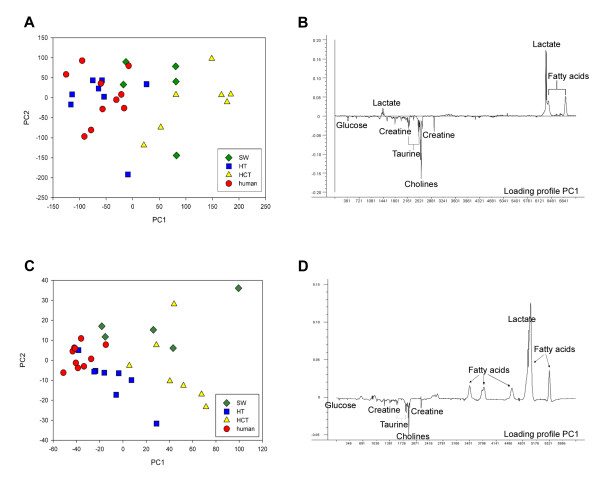
**Score plot of PC1 versus PC2 from PCA of spin-echo (A) and single pulse (C) spectra from untreated HT29, HCT116, SW620 xenografts and rectal cancer biopsies (A).** The corresponding loading profiles of PC1 are shown next to the score plots (B and D).

Score plot of the first principal component (PC1) versus PC2 from PCA of the spin-echo spectra is shown in Fig. 3A. These two components describe 65% of the total variation in the spectra. PCA demonstrates a grouping of spectra from SW620, HCT116, HT29 and human rectal cancer biopsies with the HT29 xenografts exhibiting the metabolic profile most similar to human rectal cancer biopsies. The variation in metabolic profile of human rectal cancer tissue is small as seen by the clustering of the spectra in the score plot. The loading profile of PC1 explains 40% of the total variation in the spectra. It shows that differences in relative concentrations of lipids, lactate and choline-containing compounds (Fig. 3B) are the main reason for the appearance of the clusters. Spectra with a high score value for PC1 have relatively lower levels of cholines and higher levels of lipids and lactate. There is large variation in PC2 for all xenograft species as well as for the human rectal cancer biopsies and hence, PC2 contributes minimally to clustering of the spectra. Color-coding the score plot with respect to histologically determined necrotic fractions showed that differences in necrotic fraction did not explain grouping of the spectra. Despite single-pulse spectra being dominated by lipid signals, PCA of the single-pulse spectra showed similar clustering in the score plot (Fig. 3C) as for the analysis of the spin-echo spectra (Fig. 3A). For the single-pulse spectra PC1 described 76% of the total variation in the spectra.

**Figure 4:**
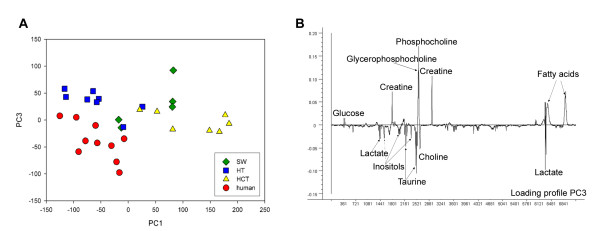
**Score plot of PC1 versus PC3 from PCA of the spin-echo spectra from untreated HT29, HCT116, SW620 xenografts and rectal cancer biopsies (A).** Loading profile of PC3 (B).

Although HT29 xenografts and human rectal cancer biopsies exhibit similar score values for PC1, their metabolic profile is not identical as seen by the score plot of PC1 versus PC3 (Fig. 4A) and the loading profile of PC3 (Fig. 4B) which describes 9% of the total variation in the spectra. Compared to HT29 xenografts, human rectal biopsies contain relatively higher amounts of lactate, taurine, inositols, choline and less of glucose, creatine, phosphocholine and glycerophosphocholine.

### Metabolic profiles of irradiated and untreated HT29 xenografts

**Figure 5:**
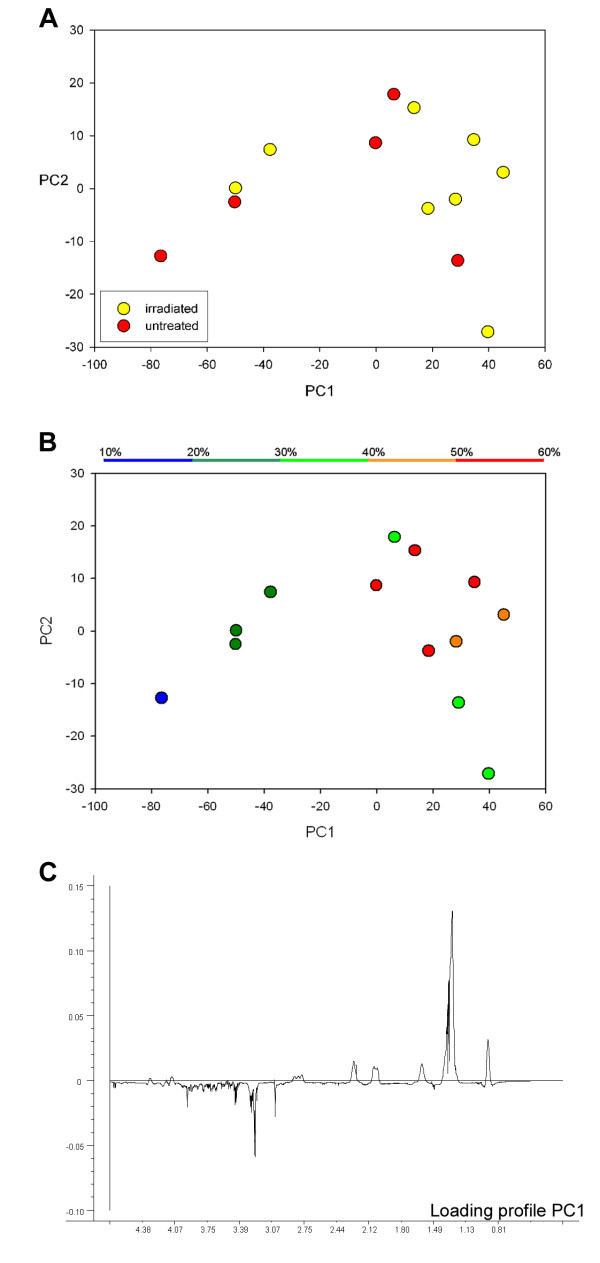
**Score plot of PC1 and PC2 from principal component analysis of the spectral region 4.7 - 0.5 ppm from spin-echo MAS MR spectra of irradiated and untreated HT29 xenografts with color coding according to untreated (red)/irradiated (yellow) (A) and amount of necrosis in the samples (B).** The corresponding loading profile of PC1 (C). Each point in (A) and (B) represents a single tumor tissue sample.

In Fig. 5 the score plot of PC1 and PC2 from PCA of single-pulse spectra (Fig. 5A and 5B) and the corresponding loading profile of PC1 (Fig. 5C) of irradiated and untreated HT29 xenografts are shown. PC1 and PC2 account for 79 and 8% of the total variation in the spectra, respectively, and the differences between spectra are mainly due to differences in relative lipid content of individual samples. Spectra from two irradiated HT29 xenografts cluster together with the untreated xenografts (Fig. 5A). Thus, a distinct separation of untreated and irradiated xenografts based on their spectral metabolic profile is not accessible. The two irradiated tissue samples grouping together with the untreated biopsies had substantially lower necrotic fractions than the other irradiated xenografts as illustrated in Fig. 5B where the score plot from Fig 5A is color-coded with respect to necrotic fraction. This indicates a strong association between necrotic fraction in the samples and their score for PC1.

**Figure 6:**
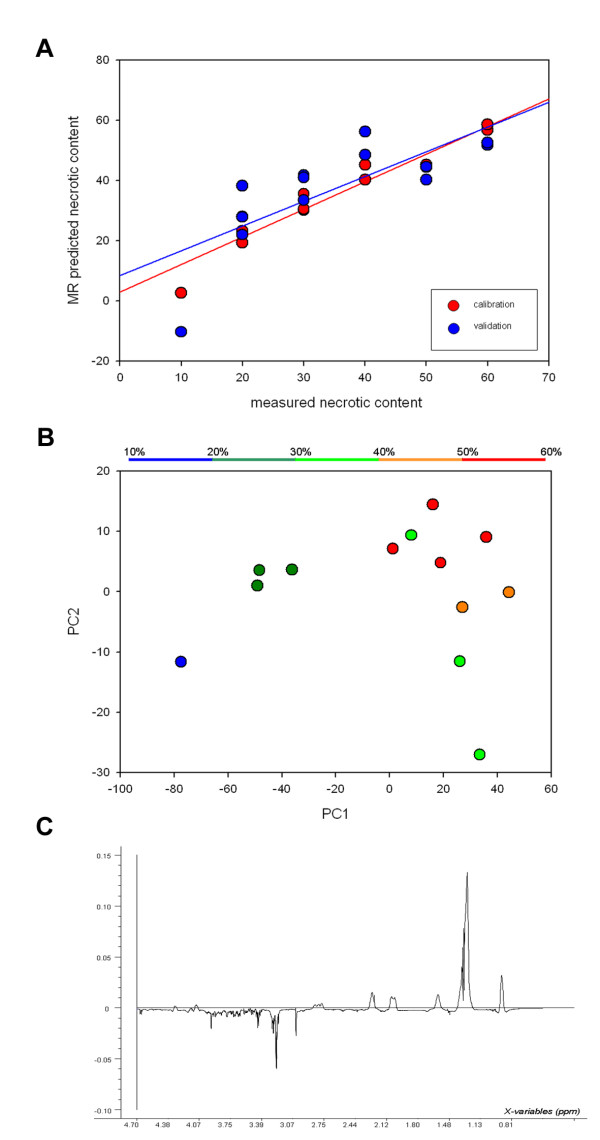
**Correlation between measured necrotic fraction and predicted necrotic fraction based on the samples metabolic profile (r = 0.96 p < 0.001 for training and r = 0.76, p < 0.002 for validation) (A).** Score plot of PC1 versus PC2 from PLS calibration of necrotic fraction to single-pulse spectral data for untreated and irradiated HT29 xenografts (B) and the corresponding loading profile of PC1 (C).

By relating single-pulse spectra to the necrotic fraction (both irradiated and untreated) using PLS, a strong correlation was found between necrotic fraction predicted by HR MAS MRS and by histology (Fig. 6A). PC1 and PC2 from PLS analysis accounted for 86% and 73% of the total x- and y-variation, respectively (Fig. 6B). The loading profile of PC1 was dominated by lipid signals and was almost identical to the loading profile of PC1 from the analysis of untreated and irradiated HT29 xenografts (Fig 6C). Thus, radiation-induced therapy response was not detectable, whereas necrotic fraction can be assessed from the spectral data; samples with high necrotic fractions had high score values for the first principal component (PC1) compared to those with low necrotic fractions (Fig. 6B). PCA and PLS of spin-echo spectra showed similar results as analysis of single-pulse spectra (no plots shown).

## Discussion

Xenografts in animals are valuable models in predicting clinical outcome of therapeutic interventions. Molecularly well-characterized xenografts may also serve as valuable tools in target-orientated drug development of specific rationally designed small molecules. Knowledge of the differences in metabolic composition between tumors arising in mice and those in humans might help selecting the appropriate xenograft model. The human HT29, HCT116 and SW620 cell lines are among the most commonly used models representing colorectal cancer [18].

To the authors knowledge this is the first HR MAS MRS analysis and assignment of metabolites in rectal cancer. The spectral information obtained by one-dimensional HR MAS MR spectra of rectal adenocarcinoma (Fig. 1D) showed resolution comparable to cell suspension [19] and perchloric acid extract [20] spectra of colorectal cancer tissue previously described in high field ^1^H-NMR. The HR MAS MR spectra were dominated by choline, lactate, inositols, creatine and taurine. The choline metabolites (choline, phosphocholine and glycerophosphocholine) are important in the phospholipids metabolism [21] and these metabolites can undergo extensive alterations resulting from malignant transformations [22-25]. For human mammary epithelial cells progression from a normal to a malignant phenotype is associated with a reversal in the ratio of phosphatidylcholine and glycerophosphocholine, as well as an overall increase in the content of these two metabolites [22].

Taurine has been shown to be elevated in rectal adenocarcinoma compared to healthy mucosa [26], and thus a potential tumor marker. Further studies are needed to assess the full potential of HR MAS MRS in the diagnosis and grading of rectal adenocarcinoma.

PCA of spectra from human rectal cancer tissue and HT29, SW620 and HCT116 xenografts showed that the HT29 xenograft had the metabolic profile most similar to rectal cancer biopsies. Nevertheless, there are differences in metabolic patterns of HT29 xenografts and human rectal cancer biopsies that could influence their usefulness as preclinical tumor models. First, the human samples had relatively higher lactate levels than the HT29 xenografts. If the elevated tumor lactate level observed is a result of hypoxic tumor environment, hypoxia-mediated radiation resistance could lead to overestimation of the therapeutic effect of chemotherapy and radiotherapy in a preclinical model [27,28]. The HT29 xenografts also exhibited higher levels of glycerophosphocholine and phosphocholine and lower levels of choline than rectal cancer biopsies suggesting an increased tumor proliferation rate and cell membrane turnover through the activation of the phosphatidylcholine pathway in the xenografts [21]. The amounts of choline metabolites differ between cell lines and for breast cancer cells it has been proposed that this distribution is linked to malignancy and metastatic potential [23,24]. Compared to human rectal cancers, the rapidly growing HT29 xenograft is poorly vascularized and associated with subsequent development of hypoxic areas and tumor aggressiveness.

Histological examination of the tissue samples revealed large differences in necrotic fraction, viable cell fraction and fibrosis between tissue samples. None of the human colorectal cancer biopsies contained necrotic tissue, whereas the necrotic fraction for the xenografts varied between 5 and 80%. The non-existence of necrosis in the human tissue samples in this study could be due to the absence of necrosis in these human rectal cancers or related to the biopsy procedure. We found no association between the metabolic profile of non-irradiated tumors and necrotic fractions, which confirms that the clustering of spectra is based on true metabolic differences and not on the amounts of necrosis within the samples.

PCA of spectra from irradiated HT29 xenografts and untreated HT29 xenografts showed that two of the irradiated samples clustered together with the untreated tumors, probably due to the high necrotic fraction in these two samples. Thus, in our study PCA could not be used to evaluate treatment induced metabolic changes. A better differentiation between viable and necrotic areas before HR MAS to ensure that viable tumor tissue is investigated will be necessary in order to detect the treatment induced metabolic changes. Well-developed HT29 xenografts have necrotic centers and an outer rim of viable cells. Selection of tissue sample from these xenografts is critical and will largely influence the results. Still, these sampling errors are similar to those associated with other clinically used invasive methods.

There was a strong correlation between necrotic fraction and the metabolic profile of untreated and irradiated HT29 xenografts. Differences in metabolic profiles were based on their relative amount of necrosis rather than radiation-induced effects. Furthermore, a significant correlation was found between predicted and measured necrotic fraction (r = 0.76, p < 0.002) (Fig. 6A), indicating that the necrotic fraction in the sample can be assessed from the samples metabolic profile.

## Conclusion

A large number of metabolite peaks were identified and assigned in the HR MAS MR spectra of human colorectal cancer and xenograft parallels. By PCA calibration, differences in the metabolic composition between HT29, HCT116 and SW620 xenografts and human rectal cancers were revealed. HT29 xenografts were found to have the metabolic profile most similar to human rectal tissue biopsies. The necrotic fraction in the xenografts affects the metabolic profile and in order to assess treatment-induced metabolic changes investigation of only viable tumor tissue should be ensured.

## Methods

### Animals and xenografts

Xenografts were generated by s.c. transplantation of human colorectal HT29 (n = 21), HCT116 (n = 7) and SW620 (n = 5) tumor tissue fragments (~2 × 2 × 2 mm^3^) into the flanks of athymic nude mice. The animals (20–30 g, 7–9 weeks old) were bred at the animal department of our institute and kept under specific pathogen-free conditions at constant temperature (22 – 24°C) and humidity (30 – 50%). The animals were given sterilized food and tap water *ad libitum*. The experiment was approved by the National Committee on Research on Animal Care and executed according to the Interdisciplinary Principles and Guidelines for the Use of Animals in Research, Marketing and Education (New York Academy of Science, New York, NY).

Eight of the HT29 xenografts were given a single dose of 15 Gy using a ^60^Co unit (Mobaltron 80, TEM Instruments, Crawley, United Kingdom) with a dose rate of 0.6 Gy/min. The animals were anesthetized with a 25 mg/kg s.c. injection of a mixture of tiletamine 2.4 mg/ml and zolazepam 2.4 mg/ml (Zoletil vet^®^, Virbac Laboratories, Carros, France), xylazine 3.8 mg/ml (Narcoxyl vet, Roche, Basel, Switzerland) and butorphanol 0.1 mg/ml (Torbugesic, Fort Dodge Laboratories, Fort Dodge, Iowa, USA). Radiation dose deposition to other parts of the animals was minimized by placing the animals in the outer corners of the 10 × 10 cm^2 ^radiation field. Five millimeter buildup bolus was placed on top of the tumors to give maximum dose deposition within the tumor volume. Termoluminescence (TLD) chips monitored the individual tumor doses.

All mice were sacrificed with neck dislocation six to nine weeks after inoculation when the mice were bearing 500 mm^3 ^to 900 mm^3 ^subcutaneous tumors. The tumors were immediately excised and put on liquid nitrogen, and further stored at -70°C until HR MAS MRS analysis.

### Rectal cancer biopsies

Tumor biopsies from 10 patients (7 men and 3 women) with histologically proven rectal adenocarcinoma (T2-4, N0-2, M0) were included in this study. The median age was 58 years (range; 43–73). Tumor biopsies were immediately frozen in liquid nitrogen, labeled and further stored at -70°C until HR MAS MRS analysis. The patients gave written informed consent to participate in the study that was approved by the local ethics committee.

### HR MAS MRS

Prior to HR MAS MRS analysis, all samples were cut (mean weight; xenografts: 15.68 mg, patient samples: 18.19 mg) to fit a MAS rotor (50 μL, 4 mm o.d.). Phosphate buffered saline (PBS, 40 μL)) in D_2_O (deuterium lock reference) containing trimethylsilyl tetradeuteropropionic acid (TSP) as a chemical shift reference was added to the sample. Excess buffer was removed in the rotor assembling procedure. Sample preparation took approximately 10 minutes and was performed on top of ice blocks.

HR MAS ^1^H MR spectra were recorded using a Bruker Avance DRX600 spectrometer equipped with a ^1^H/^13^C MAS probe that had the gradient aligned with the magic angle axis (Bruker BioSpin GmbH, Germany). Samples were spun at 5 kHz using an instrumental temperature setting of 4°C.

Two one-dimensional acquisitions were performed for all samples. First, single-pulse spectra (zgpr; Bruker) were obtained using 3.0 s of water presaturation and a 60° flip angle over a sweep width of 20 ppm. The free induction decay (FID) was acquired during 1.36 s and collected into 32 K points, giving a repetition time of 4.36 s. A total of 32 FIDs were recorded and averaged. Thereafter, Carr-Purcell-Meiboom-Gill (CPMG) spin-echo experiment (cpmgpr; Bruker) was performed using 2.0 s of water suppression prior to a 90° excitation pulse. Reduction of signal from metabolites with short T_2 _relaxation (T_2_- filtering) was obtained using a delay of 1 ms repeated 136 times resulting in a 285 ms effective echo time. One hundred twenty-eight FIDs over a frequency region of 16.7 ppm were collected into 32 K points, resulting in an acquisition time of 1.64 s. The repetition time was 3.93 s. Raw data of both single-pulse and spin-echo spectra were multiplied with a 0.3 Hz exponential line broadening before Fourier transformation and application of a second order linear baseline correction.

Homonuclear correlated spectra (COSY) were obtained using a standard pulse program including gradients and water presaturation (cosygpr, Bruker). 52 transients per increment for 512 increments were collected into 2 K data points. A spectra width of 13.3 ppm was used in both dimensions. The time domain data were zero-filled and multiplied with a sine function in both dimensions before Fourier transformation.

### Histology

Immediately after acquisition of HR MAS MR spectra all specimens were fixed in 10% formalin and embedded in paraffin. One 5 μm cross-section was cut from each block, stained with haematoxylin, erythrosine and saffron, and examined microscopically with respect to viable tissue, necrosis and fibrosis by an experienced pathologist. Analysis of variance (ANOVA, SPSS version 13.0 Science, Chicago, IL, USA) was used for comparing group means.

### Multivariate analysis

Separate matrices of single-pulse and spin-echo spectra were constructed by converting the region of interest (4.7 - 0.5 ppm) to ASCII files. These spectra were peak aligned [29,30], and imported into the Unscrambler package (CAMO process AS, Norway). Baseline offset was adjusted, and the number of points in the selected spectral region was reduced to half of its original size by averaging. Areas in the spectra containing contamination from lubricant gel (human samples) and ethanol (due to laboratory sterilizing procedures) were removed from the matrices. The remaining spectral regions were mean normalized (areas below spectra made equal).

Principal component analysis (PCA) was performed on matrices of spectra from untreated HCT116, SW620 and HT29 xenografts (n = 20) and human cancer biopsies (n = 10) for comparison of their metabolic profiles. Two-dimensional score plots and loading profiles of the principal components (PC) were used to visualize the relative contribution of individual metabolites to the clustering of the different spectra. To examine metabolic changes following irradiation, PCA was performed on matrices of spectra acquired from irradiated and untreated HT29 xenografts. The correlations between the tumors metabolic profiles and necrosis were examined by performing partial least square (PLS) regression.

All model calibrations (PCA and PLS) were performed both on matrices of spin-echo and single-pulse spectra, with mean centered data and full cross-validation (leave-one-out). In the PLS modeling, the number of PCs to retain in the model was determined by minimizing the total residual y-variance and the root mean square of prediction. Pearson's correlation analysis was used to determine the connection between MR predicted and histological measured necrotic fraction.

## Competing interests

The authors declare that they have no competing interests.

## Authors' contributions

TS, KR, TFB and BS designed the concept of this study, carried out the collection of the data, interpreted the statistical results and drafted the manuscript. BS and TFB performed the statistical analysis with interpretation. JH performed the histological analysis. KF and AHR provided biopsies from rectal cancers. DRO and ISG discussed and revised the manuscript. All authors were involved in the research presented and approved the final manuscript.
